# Cognitive reappraisal in mHealth interventions to foster mental health in adults: a systematic review and meta-analysis

**DOI:** 10.3389/fdgth.2023.1253390

**Published:** 2023-10-20

**Authors:** Karolina Morello, Sarah K. Schäfer, Angela M. Kunzler, Lilli-Sophie Priesterroth, Oliver Tüscher, Thomas Kubiak

**Affiliations:** ^1^ Leibniz Institute for Resilience Research (LIR) Mainz, Mainz, Germany; ^2^ Clinical Psychology, Psychotherapy and Diagnostics, Technische Universität Braunschweig, Brunswick, Germany; ^3^ Institute for Evidence in Medicine, Medical Center - University of Freiburg, Faculty of Medicine, University of Freiburg, Freiburg, Germany; ^4^ Health Psychology, Institute of Psychology, Johannes Gutenberg University Mainz, Mainz, Germany; ^5^ Department of Psychiatry and Psychotherapy, University Medical Center of the Johannes Gutenberg University Mainz, Johannes Gutenberg University Mainz, Mainz, Germany; ^6^ Institute of Molecular Biology (IMB), Mainz, Germany

**Keywords:** mHealth, mental health apps, ecological momentary intervention, mental health, reappraisal, cognitive restructuring, systematic review, meta-analysis

## Abstract

**Background:**

An increasing number of mHealth interventions aim to contribute to mental healthcare of which interventions that foster cognitive reappraisal may be particularly effective.

**Objectives:**

To evaluate the efficacy of mHealth interventions enhancing cognitive reappraisal to improve mental health in adult populations.

**Methods:**

The literature search (four databases) yielded 30 eligible randomized controlled trials (comprising 3,904 participants). We performed a multi-level meta-analysis to examine differences between intervention and comparator conditions at post-intervention assessment. Moderator analyses were conducted for potential moderator variables (e.g., type of comparators).

**Results:**

Most interventions were CBT-based with other training components in addition to cognitive reappraisal. We found preliminary evidence for a small to medium effect favouring mHealth interventions to enhance cognitive reappraisal over comparators, *M*(SMD) = 0.34, *p *= .002. When analysing single symptoms, there was evidence for a small to medium effect of mHealth interventions on anxiety and depressive symptoms, but not for psychological distress and well-being. All analyses showed substantial heterogeneity. Moderator analyses revealed evidence for more favourable effects in studies with passive comparators. There was an overall high risk of bias in most of the studies.

**Conclusions:**

We found preliminary evidence for a small to medium effect of mHealth interventions including a cognitive reappraisal component to improve mental health. However, most of the interventions were complex (i.e., reappraisal was provided alongside other components), which prevents us from examining reappraisal-specific effects beyond general mental health promotion in mHealth. Dismantling studies examining the effects of single intervention components are warranted to corroborate these promising results.

**Systematic Review Registration:**

https://www.crd.york.ac.uk/prospero/display_record.php?RecordID=142149, identifier [CRD42019142149].

## Introduction

The high prevalence of mental disorders is a key challenge for healthcare (1). Digital technologies such as mobile phones can potentially improve the dissemination of evidence-based mental health interventions (2, 3). Thus, numerous mobile-based programs, also referred to as mobile health (mHealth) interventions, were developed and distributed in the last years. These aim at improving mental health and well-being or reducing symptoms of mental disorders of their users. Using mobile apps, trainings can easily be delivered to users in their everyday lives and natural settings in a timely fashion (4).

mHealth apps can be implemented as stand-alone interventions, that might also be used as easy-to-access self-help programs. Alternatively, mHealth can be used as an add-on to face-to-face treatments, e.g., for therapeutic guidance (2). Thus, mHealth is an easily accessible, low-cost method that has the potential to help overcoming supply gaps in mental healthcare (5, 2, 6).

A number of clinical trials as well as recent systematic reviews and meta-analyses pointed to an increasing importance of mHealth interventions for mental health care and promotion (3, 7–12). Those studies provided evidence for mHealth interventions being feasible, acceptable, and effective in different populations and for different purposes. In line with common classifications (13, 14), mHealth interventions can be used in the following domains: First, many mHealth interventions aim at reducing symptoms of mental disorders (i.e., treatment) such as anxiety disorders and depression (15–18, 11). However, not only patients with manifest mental disorders can be targeted by mHealth interventions. For at-risk groups, subpopulations, or the general community mHealth interventions may also have the potential to prevent mental disorders [i.e., prevention (19)] or to promote positive aspects of functioning and well-being [i.e., mental health promotion (20)]. Research focuses primarily on mHealth interventions, which aim at reducing symptoms of mental disorders (21, 9), but there is some evidence on the effects of mHealth interventions for the general community (22, 20) which highlights the benefits for a broader target group.

The increasing popularity of mHealth interventions to reduce symptoms of mental illness as well as to promote mental health claims for their critical evaluation to provide users with effective and safe technologies. However, the scientific evaluation of mHealth trainings is still in its beginnings and the integration of scientifically validated theories and strategies into mHealth interventions in order to maximize a favorable mental health impact often remains unclear (23–25). For example, a recent systematic review examined the extent to which evidence-based contents are included in popular smartphone apps for the treatment of depression and anxiety (26). The study found that most of these apps included at least one evidence-based treatment element. However, specific evidence-based components were found rarely in those apps. Thus, there is evidence for a gap between empirically supported treatment components and the components used in mHealth interventions (26). Moreover, it becomes apparent that many mHealth interventions are multi-component interventions (27). They offer a range of components in combination with each other, of which some are evidence-based whereas others are not, which further complicates a statement on the effectiveness of single components.

One of the above-mentioned evidence-based intervention components is cognitive reappraisal, which pertains to modify dysfunctional thoughts. Cognitive reappraisal is an emotion regulation strategy that has been defined as creating alternative appraisals or interpretations of a potentially emotion-eliciting situation to change its emotional impact (28–30). As a strategy of cognitive change, reappraisal can be achieved through techniques of cognitive restructuring, reframing or reinterpretation, which represent key tactics of cognitive behavioral therapy [CBT (31, 32)].

Evidence comes from meta-analyses on the impact of reappraisal on mental health: Cognitive reappraisal has been found to be negatively correlated with symptoms of mental distress (33–36) and positively with indicators of mental health such as well-being (37, 35). Moreover, reappraisal appears to be associated with successful coping and maintaining of well-being when facing stressful events [i.e., resilience (38)]. Therefore, cognitive reappraisal might be a valuable ingredient for all types of mHealth interventions (treatment, prevention, or mental health promotion).

It is well established that reappraisal processes play a central role in the development and maintenance of mental illness, as changing dysfunctional cognitions is essential to reduce symptoms of mental disorders (31). Following a mechanistic approach, reappraisal might be seen as an active therapeutic mechanism. This means, that an effective psychological treatment might enhance reappraisal skills of an individual, which in turn contributes to symptom change (39). In line with this notion, there is evidence from CBT-based interventions that changes in symptom-related cognitions (i.e., reappraisal) mediate changes in symptoms, for example of panic disorder (40) and post-traumatic stress disorder [PTSD (41)].

On the other hand, research on the specific effects of reappraisal components in CBT interventions is still ongoing. Some mixed evidence is coming from research on the effectiveness of specific intervention components in complex CBT interventions. While some studies found no evidence for specific effects of any components (42) or no additive effect of reappraisal-related components (43, 44), Pompoli et al. (45) found that cognitive restructuring components are associated with the largest remission rates in panic disorder.

Notably, this research on reappraisal components does not focus the setting of mHealth interventions. Therefore, it remains unclear whether the advantages of mHealth, such as facilitating a continuous training of cognitive reappraisal in everyday life at a time, when a person needs support, can be put into practice.

Therefore, this systematic review and meta-analysis aims to examine the efficacy of psychological mHealth interventions comprising a component of cognitive reappraisal to enhance mental health in clinical and non-clinical adult populations.

## Methods

A review protocol was registered with PROSPERO (ID: CRD42019142149). The review is reported in accordance with the Preferred Reporting Items for Systematic Reviews and Meta-analysis [PRISMA (46)]. We used the web-based platforms Covidence (47) and Rayyan (48) for the screening process.

### Search strategy

A comprehensive literature search in MEDLINE (via PubMed), PsycArticles (via Ebscohost), Embase (via Ovid) and CENTRAL was conducted (search starting from the incept of each database; last update: March 7, 2022). The search process for this review integrated search terms for several higher-level resilience mechanisms identified in a landmark theory paper [(49); i.e., cognitive reappraisal, cognitive flexibility, extinction learning, interference inhibition, and stress immunization]. Of these resilience mechanisms, only cognitive reappraisal is relevant for the present review. Records addressing other resilience mechanisms than cognitive reappraisal were excluded throughout the process of study selection to ensure sufficient comparability of the included studies. The search strategy comprised four clusters of search terms that were searched in title, abstracts and (partly) in keywords: (1) cognitive reappraisal (e.g., cognitive restructuring, emotion regulation); (2) intervention (e.g., training, program); (3) mobile delivery [e.g., ecological momentary intervention (EMI), mobile application] and (4) study design [e.g., randomized controlled trial (RCT)]. Within clusters, search terms were combined using the Boolean operator OR, while clusters were linked using the operator AND. The search strategies for all databases are presented in Supplementary Material A.

### Study selection

All identified records were screened by two independent reviewers. First, clearly irrelevant records were excluded at title/abstract level. Second, eligibility was assessed at full-text level (kappa = .65), where we examined whether the mHealth interventions contained at least one reappraisal-enhancing component. For this purpose, we retrieved additional materials (i.e., study protocols, websites, app store or play store entries) related to the respective mHealth interventions, to gather information on intervention components. Discrepancies were resolved through discussion or by consulting a third author at each stage, and consensus was achieved in all cases.

### Eligibility criteria

Studies were eligible if they met the following criteria: (a) An adult study sample (≥18 years) from a clinical or non-clinical population was assessed. (b) The intervention used mobile devices (e.g., smartphones, tablets) to deliver intervention components to participants in their everyday lives. We considered stand-alone mHealth interventions as well as interventions that included a mobile component as an adjunct to other delivery formats (e.g., face-to-face). The examined interventions did have to address cognitive reappraisal and provide related techniques (e.g., cognitive restructuring; see Supplementary Material B for more information). (c) Included studies were RCTs, controlled trials (CTs), or cluster-randomized trials with a comparison of the mHealth intervention against any control group (e.g., wait-list control, no treatment control) or any other treatment not comprising a cognitive reappraisal component (e.g., face-to-face intervention, treatment as usual, other mHealth intervention). (d) The studies assessed at least one mental health outcome (primary outcome), that is, depressive symptoms and anxiety symptoms, measures of general psychological distress, and well-being outcomes (e.g., quality of life). Secondary outcome was cognitive reappraisal. We considered results of self-reported questionnaires, for example the Patient Health Questionnaire [PHQ-9 (50)] for depression, or the Cognitive Emotion Regulation Questionnaire [CERQ (51)] for cognitive reappraisal, and clinician-administered interviews. (e) Eligible studies were peer-reviewed publications published in English.

### Data extraction and coding

Two independent reviewers extracted relevant information of the included studies using a predefined data extraction sheet. The following information were extracted: (a) study details (e.g., first author, publication year, country); (b) sample characteristics (e.g., sample size, mean age, gender balance); (c) intervention characteristics (e.g., name of the mHealth intervention, intervention length, availability of human support, proportion of cognitive reappraisal); (d) study methods (e.g., design, type of comparator); (e) mental health outcomes and measures.

For all included studies, we defined relevant intervention arms and outcome measures. Arms comprising a mHealth intervention with a cognitive reappraisal component were defined as intervention group; other arms were defined as comparators. For each study, all reported outcomes of interest for these meta-analyses were considered. More details on data extraction and coding (e.g., definition of availability of human support, intervention type, intervention triggering as well as proportion of cognitive reappraisal) are presented in Supplementary Material B.

### Quality and bias assessment

#### Risk of bias of primary studies

As only RCTs were identified, risk of bias was assessed independently and in duplicate in the following domains using the Cochrane Risk of Bias Assessment Tool 2 [RoB2 (52)]: (a) bias arising from the randomization process; (b) bias due to deviations from intended interventions; (c) bias due to missing outcome data; (d) bias in measurement of the outcome; and (e) bias in selection of the reported result. With one identified crossover RCT, we proceeded according to the additional considerations for crossover trials of the RoB2 tool. In addition to bias ratings per domain, the overall risk of bias was assessed at study level. Ratings could be “low” or “high” risk of bias or express “some concern” (52). The interrater agreement was high (96.2%), and all disagreements were resolved through discussion.

#### Publication bias

The potential impact of publication bias was examined by using visual inspections of contour-enhanced funnel plots (53) as well as by means of statistical analyses by approximating the Begg Mazumdar rank correlation test (54). This test is not available for multilevel meta-analysis but can be approximated by including the sampling error as moderator variable to the main analysis. In case the sampling error would significantly predict study effect sizes, this can be interpreted as evidence of a non-normal distribution of effect sizes and thus, suggest the presence of a publication bias.

### Data synthesis

Included studies were summarized narratively and in tabular form. Pairwise meta-analyses (intervention vs. comparator) were performed for primary outcomes if at least two studies were available, and these were sufficiently homogeneous in terms of intervention types and outcomes. For studies with multiple intervention arms, it was determined which arm was most relevant for this review. In case more than one arm was relevant, these were averaged according to the recommendations of the Cochrane Collaboration (55). For our meta-analyses, we included multiple types of comparators (e.g., wait-list and active controls, face-to-face interventions). However, we examined the impact of this analytical decision by means of subgroup moderator analyses. In case data needed for effect size calculation was missing or ambiguous, study authors were contacted by the review team.

All statistical analyses were performed in *R* version 4.2.3 (56) using the package *metafor* (57). As we expected relevant between-study heterogeneity, all analyses used random-effects models and maximum likelihood estimations.

As effect size measure, we used standardized mean differences (SMDs, Hedges’ *g*) at post-intervention assessment and 95% confidence intervals (CIs) as indicators of their significance. SMDs were calculated based on the means, standard deviations (SDs), or alternative statistical information (e.g., standard error), with positive SMDs indicating a favorable effect of the intervention over the comparator condition (i.e., better mental health at post-intervention assessment). Moreover, as we expected to find heterogeneous results, 95% prediction intervals (PIs) were used to account for uncertainty of meta-analytical findings (55). PIs provide an estimate of the interval in which 95% of future observations will fall. In line with previous recommendations, PIs were calculated when 10 or more effect sizes were available per analysis (55).

Our main analysis aimed at answering the question of whether there is an effect of mHealth interventions aiming to enhance cognitive reappraisal on overall mental health. For this purpose, we used a multilevel approach nesting effect sizes of mental health indicators defined as primary outcomes within studies and outcome types [i.e., depressive symptoms and anxiety symptoms, measures of general psychological distress, and well-being (58)]. Thereby, our model allowed for correlations of effect sizes coming from the same study as well as for correlations of effect sizes coming from different studies but assessing the same outcome type (e.g., depressive symptoms).

If a study used more than one questionnaire to assess an outcome domain, for example the PHQ-9 and BDI-II (59) for depressive symptoms, we used the questionnaire that was administered most frequently across all studies in our analyses. In the few cases, where outcome data were available for more than one time point after the end of intervention, data of the post-intervention assessment was used to ensure between-study comparability.

Additional analyses aimed at answering the question of whether there is an effect of mHealth interventions aiming to enhance cognitive reappraisal on specific symptom types (e.g., depressive symptoms) or mental well-being. For these analyses, we used traditional meta-analyses for all primary outcomes.

Statistical heterogeneity was assessed using the Cochran’s *Q* statistic (60), with a significant *Q* statistic indicating the presence of heterogeneity. To further describe the amount of heterogeneity in our analyses, we used the *I*^2^ statistics (range: 0%–100%), with values of 50% and above indicating substantial heterogeneity (55). As part of our multilevel approach, we estimated heterogeneity related to between-study and between-outcome differences separately (58).

We had planned several moderator analyses in advance (see preregistration: CRD42019142149), which were also conducted due to the substantial heterogeneity as indicated by the above-mentioned indicators. All moderator analyses were performed using the multilevel approach of our primary analysis. We used meta-regressions for moderator analyses, that is, omnibus moderation tests (Q_M_) for categorical moderators (e.g., type of comparator) and to examine the impact of continuous moderators (e.g., sample mean age), with a significant Q_M_ test indicating the presence of a moderator effect. In case of categorical moderators, we obtained subgroup estimates from the multilevel model. Moderator analyses were performed for sociodemographic sample characteristics (i.e., mean sample age, and proportion of female participants), year of publication, population type (clinical vs. non-clinical population), proportion of cognitive reappraisal enhancing components, intervention duration, intervention type (treatment vs. prevention vs. mental health promotion), availability of human support, stand-alone mHealth interventions vs. combined interventions (e.g., mHealth intervention with face-to-face sessions) and comparator type (passive vs. active control condition).

Sensitivity analyses were performed based on the multilevel model used for primary analysis and examined the impact of risk of bias (only for risk of bias domains with sufficient between-study variation), the inclusion of a crossover RCT for which no ideal outcome data was available, and the impact of considering studies in which the cognitive reappraisal condition was defined as control condition by primary study authors.

## Results

**Figure 1:**
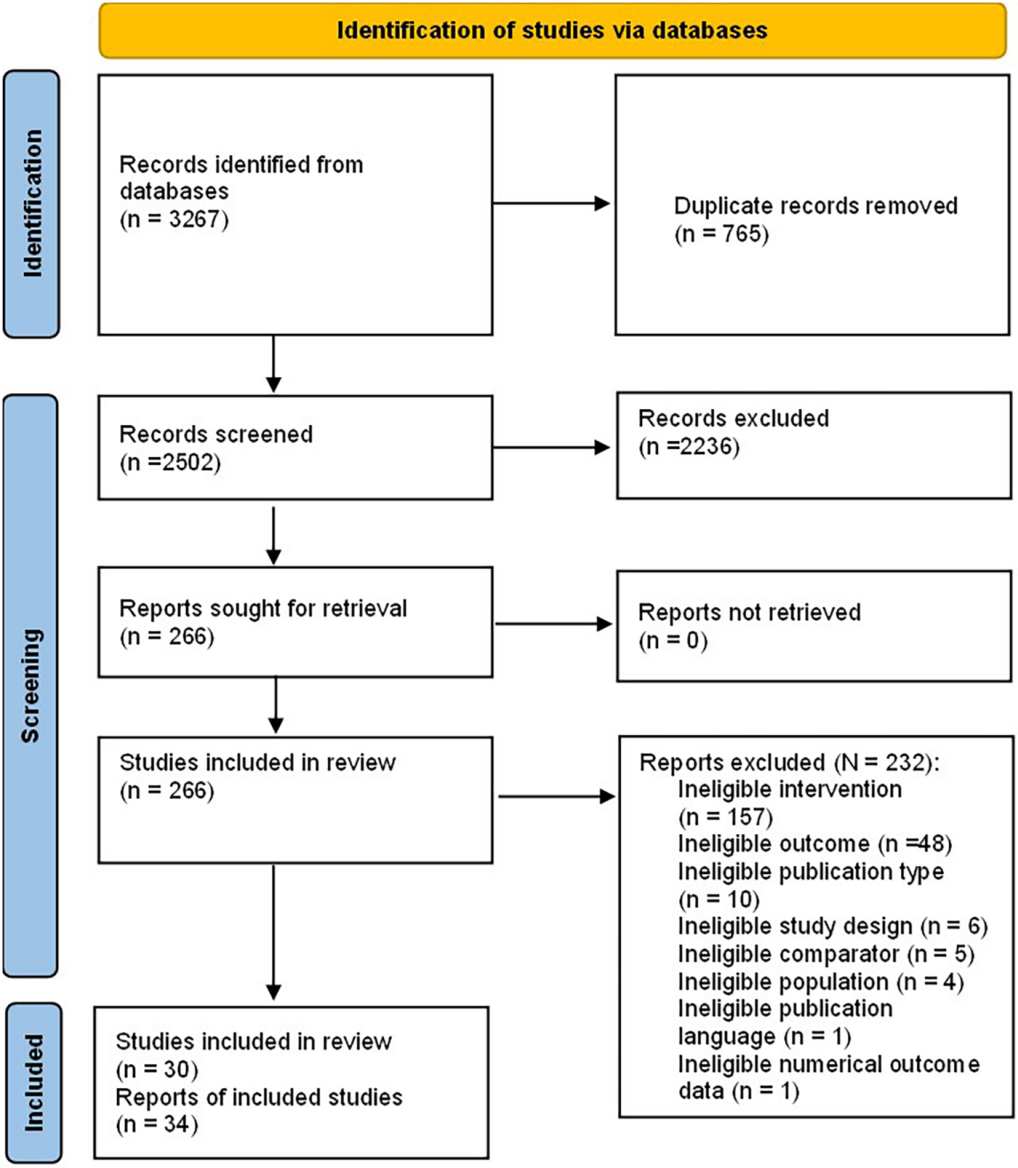
PRISMA flow diagram.

The literature search yielded 2,502 records after duplicates had been removed for title/abstract screening (see Figure 1). Of these records, 266 were assessed on the full-text level. Full-text level screenings resulted in 30 studies (from 34 reports) that were eligible for the review and included in the qualitative and quantitative summary.

### Study characteristics

Supplementary Material C provides an overview of the characteristics of the 30 studies [corresponding to 34 reports (61–95)] comprising a total of 5,377 participants at baseline (*n*_IG _= 2,697, *n*_CG _= 2,680). All studies were RCTs, including one crossover RCT (72). They were all published recently (between 2014 and 2022). Ten studies were conducted in the United States, five in Germany, three were from the Republic of Korea, two from Iran and two from China, with the remaining studies being from Canada, Australia, Japan, South Africa, Sweden, Switzerland, Vietnam, and the United Kingdom.

The mean age of the participants ranged from 18.68 to 74.93 years. The mean percentage of female participants across all studies was 68.51% (*SD* = 18.64) indicating an overrepresentation of female participants. Most studies assessed non-clinical and/or sub-clinical samples (*n* = 20), whereas samples of 10 studies were clinical (i.e., referred to as patients).

Nevertheless, also in non-clinical samples, most of the participants showed elevated symptoms of mental disorders (15 out of 20 non-clinical samples) but were not referred to as patients in a clinical setting and therefore classified as non-clinical. In total, participants of 23 out of 30 studies showed at least some elevated mental health symptoms. Sixteen studies assessed participants with depressive symptoms, nine studies reported on samples with anxiety or trauma-related symptoms, and one study examined patients with schizophrenia, schizoaffective disorder or bipolar I disorder (69). Six studies reported on samples with somatic diseases, which were the human immunodeficiency virus [HIV (94) epilepsy (61, 86), irritable bowel syndrome (72), and cancer (70, 71)]. Two studies assessed samples without any somatic or mental disease (63, 77). In line with the notion that most samples showed symptoms of mental disorders, the majority of studies (*n* = 23) mostly aimed at reducing mental symptoms. Five studies were prevention interventions and two studies focused on the promotion of mental health. Most studies primarily aimed at reducing depressive symptoms. Intervention duration ranged from 14 to 112 days (*M* = 54.57, *SD* = 26.63).

All mHealth interventions used smartphones and/or apps. For some interventions, materials could alternatively be accessed via computer or tablet, if preferred (66, 74, 86, 90). Within the mHealth interventions, the full range of multimedia materials was presented: Interventions used text-based elements combined with other materials like voice mails (77), cartoons (81, 96), interactive games (97, 90), and video material (66, 70). In addition to educational intervention elements, all studies required active engagement from the participants. Active engagement was facilitated by various types of interaction with the app or the study personnel, for example, diary entries of negative beliefs (93), multi-step questions (73, 81), rating scales (69), or games (90). Some studies were based on messenger apps (94), or integrated a chatbot (89). The majority of studies (*n *= 22) did not provide human support in addition to the mHealth intervention, while in eight studies human guidance or feedback was available. Moreover, most mHealth interventions (*n* = 23) were established in a stand-alone manner without non-mHealth components. Most studies (*n *= 14) delivered the intervention components on-demand, that is, the training was available at any time throughout the study period. Another five mHealth interventions prescribed a fixed schedule for usage (e.g., three exercises per day) and one intervention triggered participants (i.e., prompts to engage in exercises). Ten studies used a combination of on-demand, fixed and triggered delivery.

Most studies (*n* = 23) evaluated the mHealth interventions against a passive comparator (i.e., wait-list, treatment as usual, no intervention), whereas 14 studies used active comparators, with most studies using a comparable mobile app with educational material as active comparator. Seven studies assessed both, a passive as well as an active control group.

Three studies (81, 77, 69) used clinician-administered interviews for outcome assessment whereas the outcomes of the remaining studies were self-reported. Depressive symptoms were assessed in the majority of studies (*n* = 27). Nineteen studies reported on anxiety symptoms, 14 studies on well-being, and two on general psychological distress.

### Implementation of cognitive reappraisal

All interventions were theoretically based on cognitive behavioral therapy (CBT), cognitive theory (92), or cognitive behavioral stress management [CBSM, (94)]. Most of the identified mHealth interventions (*n* = 27) were multi-component interventions, meaning that cognitive reappraisal was promoted alongside other training components (e.g., behavioral activation). There were three studies (67, 73, 92) solely focusing on cognitive reappraisal. Supplementary Material D gives an overview of the components of each study as well as the relative importance of the cognitive reappraisal component in relation to the full intervention. The proportion of cognitive reappraisal in the interventions was relatively low (*M* = 28.27%, *SD* = 25.21).

### Study quality

#### Risk of bias

**Figure 2:**
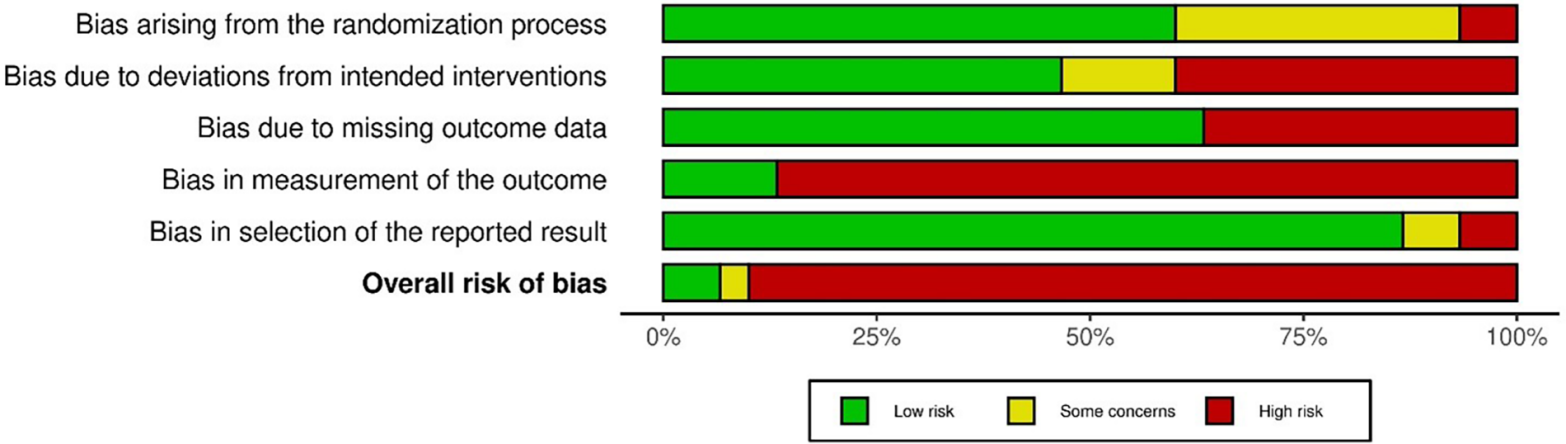
Distribution of risk-of-bias ratings within each of five bias domains and overall risk of bias according to the Cochrane risk-of-bias tool for randomized trials (RoB2) (52). All percentages are related to 30 studies (62 effect sizes) included in the review.

There was an overall high risk of bias in 27 of 30 studies (see Figure 2 and Supplementary Material E). Main flaws (≥20% some concerns or high-risk ratings) across the included studies and outcomes came from outcome measurement (89.7%, i.e., most studies used self-report measures for outcome assessment and participants were not blinded), deviations from intended interventions (53.3%), and missing outcome data (36.7%). For the study with a crossover design (72), risk of bias arising from period and carryover effects was low.

#### Publication bias

**Figure 3:**
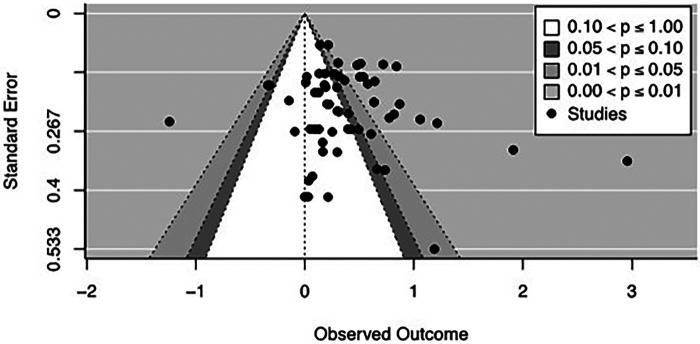
Contour-enhanced funnel plot of the main analysis. Contour-enhanced funnel plots are used to highlight significance levels of each effect size in the plot and allow to examine whether studies are missing in specific areas of the plot, that is, the non-significant areas or findings pointing to the opposite direction. For the present analysis, the plot points to a mild publication bias with a relevant number of studies being at the significant side of the right border region between the non-significant and significant area.

The meta-regression model provided evidence for a trend towards an association of sample errors and effect size estimates for the main analysis, Q_M_(1) = 3.20, *p* = .074, that is, the effect sizes are likely to violate the assumption of a normal distribution. The visual inspection of the contour-enhanced funnel plot suggested the presence of a mild publication bias (see Figure 3), with effect sizes being more likely to fall into the right significant border area of the funnel plot, while the number of studies in non-significant areas was smaller.

### Pairwise meta-analysis—intervention effect

**Figure 4:**
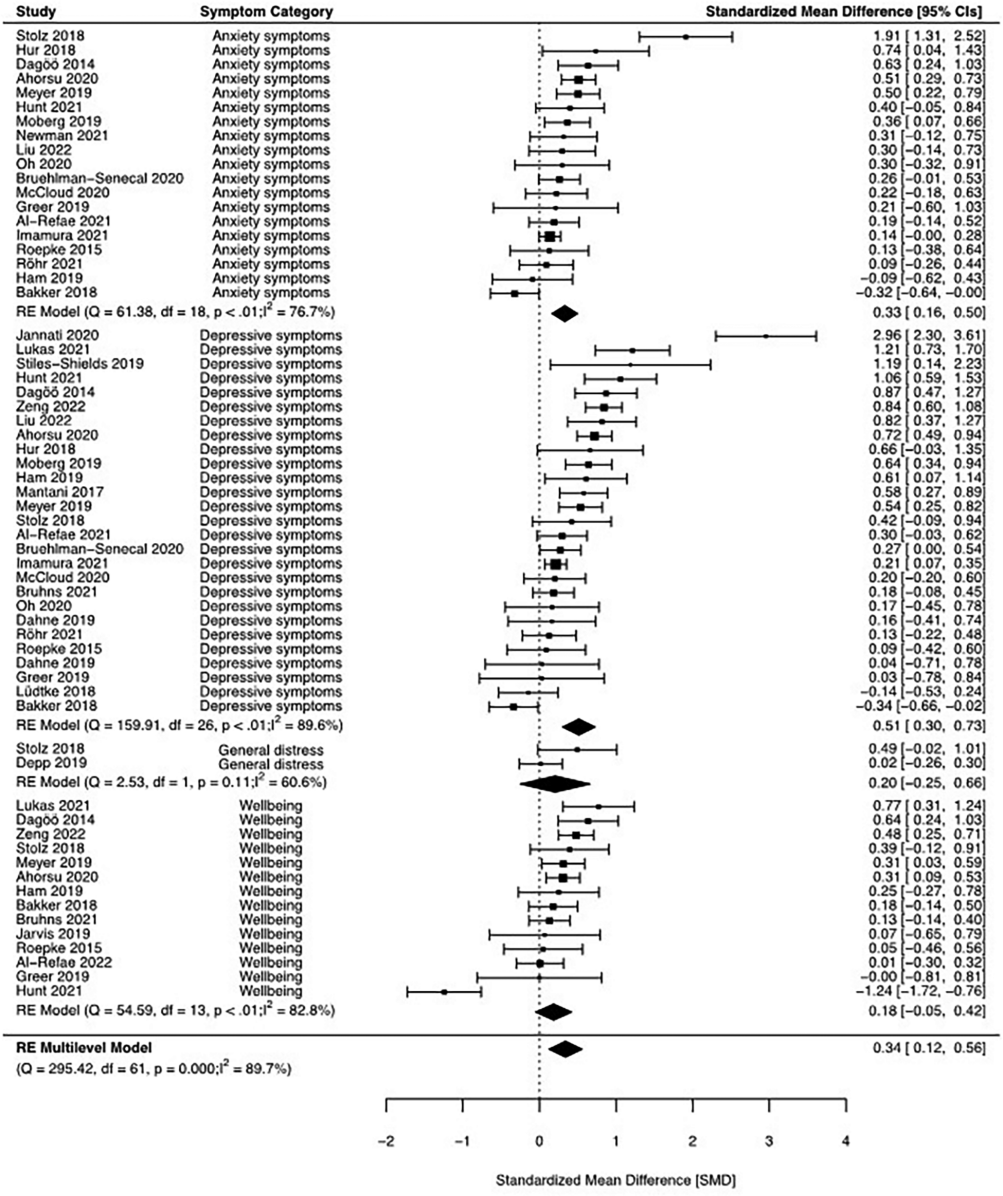
Forest plot of the multilevel meta-analysis (main analysis) comprising all mental health outcomes and traditional meta-analyses on single outcome categories. Positive effect size estimates indicate favorable effects of an intervention (i.e., reductions of mental symptoms or increases in well-being). CI, confidence interval; DF, degree of freedom; *I*^2^, heterogeneity index in percentage (range: 0%–100%); Q, Cochran’s *Q* statistic with *p* value.

Thirty studies [comprising 62 effect sizes from 9,458 observations of 3,904 participants (intervention: 4,692 observations from 1,881 participants; comparators: 4,766 observations from 2,023 participants)] were included in our quantitative synthesis (see Figure 4). Using a multilevel approach, we compared post-intervention means across all mental health outcomes between mHealth interventions to enhance cognitive reappraisal and any comparator condition. Moreover, we examined group differences of post-intervention means for single mental health indicators (see Table 1). Across all mental health outcomes, we found evidence for a small to medium effect favouring mHealth interventions to enhance cognitive reappraisal over comparators, *M*(SMD) = 0.34, 95% CI [0.12, 0.56], *p* = .002, with considerable heterogeneity between studies and mental health outcomes, Q(61) = 295.42, *p* < .001; *I*^2^ = 86.5%. However, the majority of the total heterogeneity was accounted for by between-study differences (*I*^2^ = 78.5%), while only 8.0% originated from between-outcome differences. The presence of considerable heterogeneity was further supported by the wide 95% PI covering small to medium adverse and large favourable intervention effects.

**Table 1:** Results of main analyses (multilevel and traditional meta-analyses) for primary outcomes comparing mHealth interventions to enhance cognitive reappraisal with comparator conditions.

	* *	* *	* *	Confidence interval		Prediction interval		* *	* *	* *	* *	* *
Analysis	*n*	*k*	*M*(SMD)	95%_l_	95%_u_	95%_l_	95%_u_	*p*	Q	df	*p(Q)*	*I* ^2^ *(I* ^2^ *_S,_ I* ^2^ *_O_)*
Mental health (ML)	30	62	0.34	0.12	0.56	−0.54	1.23	.002	295.42	61	<.001	86.5 (78.5, 8.0)
Anxiety symptoms (T)	19	19	0.33	0.16	0.50	−0.31	0.96	<.001	61.38	18	<.001	76.7
Depressive symptoms (T)	27	27	0.51	0.30	0.73	−0.53	1.55	<.001	159.91	26	<.001	89.6
Psychological distress (T)	2	2	0.20	−0.25	0.66	–	–	.380	2.54	1	.111	60.6
Well-being (T)	14	14	0.18	−0.05	0.42	−0.62	0.99	.126	54.59	13	<.001	82.8

The multilevel meta-analysis on mental health indicators included anxiety symptoms, depressive symptoms, psychological distress, and well-being. For all outcomes, SMDs were calculated in a way that positive values indicate favorable effects of an intervention (i.e., reductions of mental symptoms or increases in well-being).

Df, degrees of freedom; *I*^2^, heterogeneity index in percentage (range: 0%–100%); *I*^2^*_S_*, heterogeneity accounted for by between-study differences; *I*^2^*_O_*, heterogeneity accounted for by between-outcome differences; *k*, number of effect sizes; ML, multilevel meta-analysis, *n*, number of studies; Q, Cochran’s *Q* statistic with *p* value; SMD, standardized mean difference; T, traditional meta-analysis; 95%_l_, lower boundary of the 95% confidence/prediction interval; 95%_u_, upper boundary of the 95% confidence/prediction interval.

When analysing single symptoms, there was evidence for a small to medium effect of mHealth interventions to enhance cognitive reappraisal on anxiety symptoms, *M*(SMD) = 0.33, 95% CI [0.16, 0.50], *p* = .001, and depressive symptoms, *M*(SMD) = 0.51, 95% CI [0.30, 0.73], *p* = <.001. However, there was no evidence in favour of mHealth interventions to enhance cognitive reappraisal for psychological distress, *M*(SMD) = 0.20, 95% CI [−0.25, 0.66], *p* = .380, and well-being, *M*(SMD) = 0.18, 95% CI [−0.05, 0.42], *p* = .126. Overall, between-study heterogeneity was considerable for all analyses (*I*^2^ ≥ 76.7%) except for the meta-analysis on psychological distress (*I*^2^ = 60.6%), however, this was based on only two studies.

### Moderator analyses

Moderator analyses were performed for the main analysis comprising all mental health outcomes.

#### Sociodemographic variables

We found no evidence for a moderating effect of mean sample age, Q_M_(1) = 1.34, *p* = .247, and proportion of female participants per sample, Q_M_(1) = 0.15, *p* = .698.

#### Year of publication

We found no evidence for an association of publication year and effect size estimates, Q_M_(1) = 0.01, *p* = .907, that is, more recent mHealth interventions were not associated with larger effects.

#### Population type

We found no evidence for differences between intervention effects in clinical (i.e., patients) and non-clinical populations, Q_M_(1) = 0.27, *p* = .602 (see Table 2). Further, there was no evidence for differences in effects between populations with elevated symptoms of mental disorders compared to populations without symptoms of mental disorders (See Supplementary Material F).

**Table 2:** Results of the moderator analyses for categorical moderators.

Analysis	*n*	*k*	*M*(SMD)	95%CI_l_	95%CI_u_	*p*
Population type						
Clinical populations	10	22	0.28	−0.04	0.60	.083
Non-clinical populations	20	40	0.37	0.12	0.63	.004
Q_M_(1) = 0.27, *p* = .602						
Intervention Type						
Treatment	23	45	0.39	0.12	0.65	.004
Prevention	5	11	0.19	−0.25	0.64	.397
Q_M_(1) = 0.73, *p* = .394						
Availability of human support						
Human support available	8	16	0.50	0.15	0.85	.005
No human support available	8	22	0.28	0.03	0.52	.026
Q_M_(1) = 1.36, *p* = .243						
Stand alone vs. combined						
Stand alone	7	9	0.37	0.14	0.61	.002
Add-on	23	53	0.23	−0.16	0.61	.254
Q_M_(1) = 0.49, *p* = .484						
Control Condition						
Active comparator	14	30	0.13	−0.12	0.39	.316
Passive comparator	23	45	0.44	0.21	0.68	<.001
Q_M_(1) = 11.90, *p* = .001						

Results for the categorical moderator variables in the multilevel model including all outcomes, i.e., anxiety symptoms, depressive symptoms, psychological distress, and well-being. Again, positive SMDs indicate positive intervention effects. We present results of omnibus moderator tests (i.e., Q_M_ test) along with estimates per subgroup (obtained from the multilevel model). To note, for the moderator analysis on control condition, we included all effect sizes reporting on active and passive comparators separately (nested within studies), thus, the number of effect sizes included in this analysis exceeds the number of effect sizes included in the main analysis. Further, the subgroup mental health promotion contains only two studies and was left out from the analysis on intervention type. Therefore, the number of studies in the moderator analysis is lower than in the main analysis.

Q_M_(df), omnibus moderator test with degrees of freedom; *k*, number of effect sizes; *n*, number of studies; SMD, standardized mean difference, 95% CI_l_, lower boundary of the 95% confidence interval; 95% CI_u_, upper boundary of the 95% confidence interval.

#### Intervention type

As not enough studies in the category mental health promotion (*n* = 2) were available, we performed the moderator analysis only for treatment vs. prevention. We found no evidence for differences between intervention effects in treatment and prevention interventions, Q_M_(1) = 0.73, *p* = .394 (see Table 2).

#### Proportion of cognitive reappraisal component

We found no evidence for interventions comprising a higher proportion of cognitive reappraisal to be associated with larger intervention effects, Q_M_(1) = 0.02, *p* = .896.

#### Intervention duration

We found no evidence for more intense interventions, that is, interventions with more days, being associated with larger intervention effects, Q_M_(1) = 0.39, *p* = .533.

#### Availability of human support

There was no evidence for a moderator effect of the availability of human support, Q_M_(1) = 1.36, *p* = .243 (see Table 2).

#### Stand-alone versus combined interventions

Also when comparing stand-alone mHealth interventions with combined interventions (e.g., mHealth intervention with face-to-face sessions), we found no evidence for a moderator effect, Q_M_(1) = 0.49, *p* = .484 (see Table 2).

#### Control condition

There was evidence for a moderator effect of the type of comparator condition (i.e., passive vs. active comparators) Q_M_(1) = 11.90, *p* = .001. While studies using passive comparators provided evidence for a small to medium favourable effect of interventions with a cognitive reappraisal component, there was no evidence for a favourable effect when active comparators were used (see Table 2).

### Sensitivity analyses

#### Risk of bias

We re-estimated our main analysis on mental health outcomes excluding effect sizes rated to be at least “at some concern” for risk of bias in different domains (see Supplementary Material E for detailed results). Excluding all effect sizes being at least at “some concern” regarding the randomization process, the analysis yielded similar results, *M*(SMD) = 0.42, 95% CI [0.13, 0.70], *p* < .001. The same applied to analyses excluding effect sizes with at least some concerns in the domains of deviation from intended intervention, missing outcome data, and selective reporting. An analysis based on eight effect sizes at low risk for bias for outcome assessment provided no evidence for a favourable effect of mHealth interventions to enhance cognitive reappraisal, *M*(SMD) = 0.23, 95% CI [−0.12, 0.59], *p* = .202 (see Table 3).

**Table 3:** Results of main analysis excluding effect sizes rated to be at least “at some concern” for specific risk of bias domains.

	* *	* *	* *	Confidence interval		Prediction interval		* *	* *	* *	* *	* *
Risk of bias domain	*n*	*k*	*M*(SMD)	95%_l_	95%_u_	95%_l_	95%_u_	*p*	Q	df	*p(Q)*	*I* ^2^ *(I* ^2^ *_S,_ I* ^2^ *_O_)*
Randomization	18	38	0.42	0.13	0.70	−0.70	1.53	.004	195.24	37	<.001	92.1 (89.8, 2.4)
Deviations from intended intervention	14	31	0.47	0.10	0.84	−0.74	1.68	.014	186.22	30	<.001	94.2 (84.4, 9.8)
Missing outcome data	19	39	0.42	0.11	0.74	−0.75	1.60	.009	245.10	38	<.001	93.1 (86.7, 6.5)
Outcome measurement	4	8	0.23	−0.12	0.59	–	–	.202	53.95	7	<.001	86.4 (86.4, 0)
Selective reporting	26	55	0.35	0.10	0.59	−0.53	1.00	.005	285.21	54	<.001	61.5 (55.9, 5.6)

The multilevel meta-analysis on mental health included anxiety symptoms, depressive symptoms, psychological distress, and well-being. Effect-sizes that were at least ‘at some concern’ for the respective bias domain were excluded from these analyses. For all outcomes, SMDs were calculated in a way that positive values indicate favorable effects of an intervention (i.e., reductions of mental symptoms or increases in well-being).

Df, degrees of freedom; *I*^2^, heterogeneity index in percentage (range: 0%–100%); *I*^2^*_S_*, heterogeneity accounted for by between-study differences*; I*^2^*_O_*, heterogeneity accounted for by between-outcome differences; *k*, number of effect sizes; ML, multilevel meta-analysis, *n*, number of studies; Q, Cochran’s *Q* statistic with *p* value; SMD, standardized mean difference; T, traditional meta-analysis; 95%_l_, lower boundary of the 95% confidence/prediction interval; 95%_u_, upper boundary of the 95% confidence/prediction interval.

#### Crossover RCT

We included a crossover RCT in our main analysis for which no ideal outcome data was available. However, in- and excluding this trial left our results largely unchanged, *M*(SMD) = 0.37, 95% CI [0.17, 0.56], *p* < .001, as evidenced by overlapping confidence intervals.

#### Condition labelling in primary studies

In a few cases, we included conditions as intervention group that were labelled as control conditions by primary study authors. Excluding these studies from our main analysis left our results unchanged, *M*(SMD) = 0.36, 95% CI [0.14, 0.58], *p* = .002.

## Discussion

### Main findings

With this systematic review and meta-analysis, we aimed to evaluate the efficacy of mHealth interventions comprising a component of cognitive reappraisal to enhance mental health. mHealth interventions are popular and an increasing number of programs aiming at reducing mental symptoms, preventing mental disorders, or promoting mental health become available for users. Nevertheless, the scientific evaluation of effective components is still in its beginnings. Therefore, we aimed to contribute to close this gap. We followed a mechanistic approach assuming that cognitive reappraisal, as an evidence-based therapeutic mechanism, might be a particular promising target of mHealth interventions.

During the process of study selection, it became evident that cognitive reappraisal is usually included in multi-component mHealth interventions. This means that reappraisal is one of many mHealth intervention components (e.g., behavioral exercises, psychoeducation). Only three of 30 identified studies exclusively focused on the promotion of reappraisal. Thus, this review and meta-analysis cannot provide evidence on the effects of single-component reappraisal mHealth interventions as initially intended. However, the present review provided an evidence synthesis on complex mHealth interventions including a reappraisal component.

In our main analysis, we included 62 effect sizes of 30 studies. We found evidence for a small to medium overall effect favoring mHealth interventions to enhance cognitive reappraisal over comparators across all mental health outcomes. Analyses considering single symptoms (i.e., anxiety, depression, psychological distress, and well-being) revealed a more differentiated picture. Small to medium effects in favor of mHealth interventions were found for depressive and anxiety symptoms, but there was no evidence for an effect on well-being and general psychological distress. Notably, we identified only two studies with outcomes of general psychological distress. This means that mHealth interventions might be beneficial for the reduction of depressive and anxiety symptoms only and do not succeed in the improvement of positive indicators of mental health, such as well-being.

The detected effect sizes are comparable to recent meta-analyses that found evidence for small to medium effects of mHealth interventions on depression (15, 17) and anxiety (16).

Even though the meta-analysis revealed a significant overall effect of complex mHealth interventions including a cognitive reappraisal component on mental health, moderator analyses provided a more nuanced picture with favorable effects only being found when interventions were compared with passive but not active controls. In line with other meta-analyses of mHealth interventions (15, 16), this result suggests that the use of a digital device itself may provide psychological benefits. In this regard, the concept of a digital placebo effect has been proposed (98). Thus, the favorable overall intervention effect may reflect the rather unspecific effect of mHealth interventions.

We also investigated various other moderator variables (e.g., sociodemographic variables, availability of human support), but none of them reached significance. This indicates that complex mHealth interventions including a cognitive reappraisal component may be applicable to a broad range of users and a variety of intervention features.

Notably, we did not find evidence for mHealth interventions including cognitive reappraisal to be more effective when used to reduce mental symptoms compared to mHealth programs with a preventive focus. However, only a small amount of mHealth interventions with a focus on the prevention of mental disorders or mental health promotion were eligible for our systematic review. This is in line with findings from similar fields showing a strong focus on symptom reduction with limited evidence available for mental health promotion and prevention programs (99). Mostly, mHealth interventions targeted individuals with (at least) elevated symptoms of mental disorders, meaning that cognitive reappraisal was used for symptom reduction. The effectiveness of cognitive reappraisal components in mHealth interventions for more general population samples (i.e., prevention of mental disorder or mental health promotion) need further research. This ties in with an overall need for intensified research in the areas of mental health promotion and prevention of mental disorders (100).

In contrast to our expectations, we found no evidence for mHealth interventions with larger proportions of reappraisal promotion being associated with larger intervention effects. Consequently, we did not find evidence for a dose-response relationship between reappraisal proportion and intervention effects. First, this finding might be explained by the small proportion of cognitive reappraisal in most of the interventions (70% of the mHealth interventions included 20% or less reappraisal training). Second, we calculated the relation of the reappraisal-component relative to the total number of intervention components as the best available proxy for the intensity of reappraisal training. However, this might only insufficiently capture the intensity of reappraisal training. For example, a mHealth intervention containing one module on cognitive reappraisal next to a second module has a reappraisal-proportion of 50%. However, having less training modules might not automatically result in a more intense, and in turn, effective reappraisal training. Insufficient reporting of intervention details in the included primary studies prevented a more elaborated analysis.

The absence of a specific effect of cognitive reappraisal components might be interpreted with regard to the usefulness of cognitive reappraisal itself. Some authors have started to question the usefulness of cognitive reappraisal for regulating negative emotions in comparison to other emotion regulatory and cognitive mechanisms in the past (101, 102). For example, an individual participant data component network meta-analysis (cNMA) of internet-based CBT trials for depression resulted in no additive effect of cognitive restructuring components (43).

### Limitations

Our review should be interpreted in the light of its limitations. First, the included studies are limited to interventions that are provided via mobile devices such as smartphones. Therefore, we can only draw conclusions on the efficacy of cognitive reappraisal in the specific setting of mHealth and one should not generalize the findings to, for example, any digital intervention or face-to-face settings. This implies that the small effects of complex mHealth interventions including a cognitive reappraisal component might be not because of the impact of reappraisal itself but arise from problems with the mobile delivery. A training of cognitive reappraisal might be better addressed in face-to-face settings than in mHealth interventions, for example because the commitment to the intervention and participants’ willingness to engage in cognitive, sometimes challenging exercises might be more successful when supported and encouraged by a human being. Even if the use of mobile devices offers novel possibilities for encouragement and individualization, smartphones may sometimes fail to reach the individual and to consolidate reappraisal skills as a long-term engagement is often lacking. However, we cannot make a statement on the participants’ compliance in the included mHealth interventions as data on compliance were provided rarely in the studies and were not eligible for analyses.

Moreover, during the selection process, it became apparent that many studies failed to report sufficient intervention details (i.e., training content). We can therefore not rule out that we missed mHealth interventions that have used cognitive reappraisal training but failed to report it explicitly. Vice versa, we might have overrated reappraisal in some studies because we classified studies as reappraisal intervention even if the reappraisal training was part of a complex intervention. Studies might also be heterogeneous regarding their reappraisal training components in terms of schedule and content. We might have also missed eligible studies that are ongoing or unpublished as we focused the literature search on electronic databases and did not check for grey literature or perform citation searching. Moreover, our literature search ended in March 2022 and was not updated, which might have resulted in some publications being not included in our analyses. Thus, future systematic reviews will have to update our findings based on a larger number of primary studies.

Further, there are limitations that originate from the included studies. The reviewed studies were mainly multi-component interventions, which might include active intervention components next to cognitive reappraisal. Thus, the reviewed studies did not allow us to draw conclusions about a reappraisal-specific intervention effect. In addition, information whether participants acquired reappraisal skills are lacking. We could not analyze intervention effects on reappraisal because the included studies did not evaluate reappraisal-specific outcomes. Thus, we cannot answer the question of whether the included interventions in fact enhanced cognitive reappraisal.

The risk of bias assessment points towards a high risk of bias in most of the included studies. This is due to the fact that outcomes in the studies were assessed using self-report measures and participants were aware of group assignment.

### Implications and future research

Even in the light of the limitations outlined above, our findings have important implications for future research on mHealth interventions. First, more research evaluating the effects of reappraisal training via mHealth interventions are required. These are mHealth interventions consisting of an isolated training of cognitive reappraisal as well as trials with cognitive reappraisal specific outcomes. Moreover, it is important to examine whether cognitive reappraisal or its combination with other intervention components are superior in the promotion of mental health. A promising approach are dismantling studies, in which full mHealth interventions are experimentally compared with a disentangled variation of the same intervention that omits cognitive reappraisal. These studies can lead to evidence on the specific effects of single intervention components, i.e., cognitive reappraisal, and advance the research on the mechanisms of therapeutic change in mHealth interventions. Second, research on the effects and usability of mHealth interventions enhancing cognitive reappraisal skills in the real-world (i.e., effectiveness studies) should be expanded. Third, meta-analyses on individual participant data could consider Level 1-moderators (e.g., age, symptom severity) with greater statistical power, which may allow for future tailoring of interventions.

## Conclusion

Our findings provide insights into the implementation and use of cognitive reappraisal training modules in mHealth interventions. Training components related to the enhancement of cognitive reappraisal have gained scientific interest in the last years and are implemented in apps and other mHealth formats for various population groups. As expected, it turned out that most of the identified mHealth interventions were multi-component interventions, in which cognitive reappraisal was promoted alongside multiple other components. We found first evidence for a small to medium favorable effect of these complex mHealth interventions that included a component of cognitive reappraisal on mental health. The favorable effects were found only when mHealth interventions were compared with passive but not active controls. There was also no evidence for a dose–response effect of reappraisal. Thus, our findings suggest that favorable effects may arise mainly from unspecific beneficial effect of mHealth interventions. Consequently, high-quality dismantling studies examining the effects of single intervention components are warranted to corroborate and further evaluate active therapeutic mechanisms in mHealth interventions such as cognitive reappraisal.

## Data Availability

The raw data supporting the conclusions of this article will be made available by the authors, without undue reservation.

## Supplementary material

Supplementary Material A Supplementary Material B Supplementary Material C Supplementary Material D Supplementary Material E Supplementary Material F Full search strategy. Coding. Study characteristics. Proportion of cognitive reappraisal. Study quality assessment. Moderator analysis.
