# Supplementary material for: Cognitive reappraisal in mHealth interventions to foster mental health in adults: a systematic review and meta-analysis
Source: Front Digit Health. 2023 Oct 20;5:1253390. doi: 10.3389/fdgth.2023.1253390 (PMC10623449; doi:10.3389/fdgth.2023.1253390)
Supplement: Supplementary Material A Supplementary Material B Supplementary Material C Supplementary Material D Supplementary Material E Supplementary Material F — Full search strategy. Coding. Study characteristics. Proportion of cognitive reappraisal. Study quality assessment. Moderator analysis. [file Datasheet1.zip › A) Full search strategy.DOCX]

**Supplementary Material A.** Search Strategy.

The search strategy was designed for a broader search to identify various resilience mechanisms in mHealth interventions. In this meta-analysis only mHealth interventions addressing cognitive reappraisal were included from the identified records.

|  | PubMed Query |
| --- | --- |
| #1 | ((Psychological resilience[mh] OR resilience[tiab] OR Psychological adaptation[mh])) |
| #2 | ((Cognitive therapy[mh] OR CBT[tiab] OR (cognit*[tiab] AND behav*[tiab] AND therapy[tiab]) OR „cognitive training“)) |
| #3 | (((cognit* [tiab] AND (apprais*[tiab] OR reapprais*[tiab] OR refram*[tiab] OR re-fram*[tiab] OR modificat*[tiab] OR restructur*[tiab])) OR ((Emotion*[tiab] OR affect*[tiab]) AND (regulat*[tiab] OR dysregulat*[tiab])) OR Coping[tiab])) |
| #4 | ((„reward sensitivity“[tiab] OR ((positiv*[tiab] OR interpret*[tiab] OR cognit*[tiab] OR attribut*[tiab]) AND bias*[tiab]))) |
| #5 | ((((cognit*[tiab] OR affect*[tiab] OR behavior*[tiab]) AND flexibil*[tiab]) OR (task[tiab] AND switch*[tiab]))) |
| #6 | (((extinction[tiab] AND learn*[tiab]) OR (fear[tiab] AND (condition*[tiab] OR learn*[tiab])) OR extinction, psychological[mh])) |
| #7 | (((extinction[tiab] AND learn*[tiab]) OR (fear[tiab] AND (condition*[tiab] OR learn*[tiab])) OR extinction, psychological[mh])) |
| #8 | ((((Stress[tiab] OR stressor[tiab]) AND (immunization[tiab] OR inoculation[tiab] OR control[tiab] OR controllability[tiab])) OR „learned helplessness“ OR „situation control“)) |
| #9 | (attentional bias[mh] OR (attention*[tiab] AND (bias*[tiab] OR control[tiab]))) |
| #10 | #1 OR #2 OR #3 OR #4 OR #5 OR #6 OR #7 OR #8 OR #9 |
| #11 | ((train*[tiab] OR interven*[tiab] OR prevent*[tiab] OR therap*[tiab] OR program*[tiab] OR treat*[tiab] OR app[tiab])) |
| #12 | ((Ecological Momentary assessment[mh] OR (Ecological[tiab] AND momentary[tiab]) OR Smartphone[mh] OR Smartphone[tiab] OR Telemedicine[mh] OR Just-in-time adaptive[tiab] OR Mobile Application[mh] OR Cell phone[mh] OR mhealth[tiab] OR m-health[tiab] OR „mobile health“)) |
| #13 | #10 AND #11 AND #12 |
| #14 | ((randomized controlled trial[pt] OR controlled clinical trial[pt] OR randomized[tiab] OR placebo[tiab] OR drug therapy[sh] OR randomly[tiab] OR trial[tiab] OR controlled[tiab] OR groups[tiab] NOT (animals [mh] NOT humans [mh]))) |
| #15 | #13 AND #14 |

[mh] = MeSH Terms

[tiab] = Title/Abstract

[pt] = Publication Type

[sh] = MeSH subheadings

|  | Query PsycArticles (via Ebscohost) |
| --- | --- |
| S1 | MA Resilience, Psychological OR AB resilience OR MA Adjustment |
| S2 | MA (cognitive therapy OR cognitive behavior therapy) OR AB cbt OR AB ( cognit* AND behav* AND therapy ) OR TX "cognitive Training" |
| S3 | AB cognit* AND AB (apprais* OR reapprais* OR refram* OR re-fram* OR modificat* OR restructur* ) OR AB ( emotion* OR affect* ) AND AB ( regulat* OR dysregulat* ) OR AB coping OR MA (coping behavior OR emotional regulation) |
| S4 | AB "reward sensitivity" OR AB ( positiv* OR interpret* OR cognit* OR attribut* ) AND AB bias* OR MA cognitive bias |
| S5 | AB ( cognit* OR affect* OR behavior* ) AND AB flexibil* OR AB ( task AND switch* ) OR MA cognitive flexibility |
| S6 | AB (extinction AND learn*) OR AB (fear* AND AB ( condition* OR learn*)) OR MA Extinction (Learning) OR MA avoidance conditioning |
| S7 | AB (interference AND inhibit*) OR AB (response inhibit*) OR MA Executive Function OR MA interference (learning) OR TX "inhibitory control" |
| S8 | AB ( Stress OR Stressor ) AND AB ( immunization OR inoculation OR control OR controllability ) OR TX ( "learned helplessness" OR "situation control" ) |
| S9 | MA attentional bias OR AB (attention* AND bias* ) OR AB ( attention* AND control ) |
| S10 | S1 OR S2 OR S3 OR S4 OR S5 OR S6 OR S7 OR S8 OR S9 |
| S11 | AB (train* OR interven* OR prevent* OR therap* OR program* OR treat* OR app) |
| S12 | TX (ecological AND momentary) OR MA (ecological momentary assessment OR smartphones OR telemedicine OR mobile applications OR Mobile Phones OR Mobile health) OR AB ( smartphone OR “just-in-time adaptive” OR "Cell Phones" OR mhealth OR m-health) OR TX "Mobile health" |
| S13 | AB (ecological AND momentary) OR MA (ecological momentary assessment OR smartphones OR telemedicine OR mobile applications OR Mobile Phones OR Mobile health) OR AB ( smartphone OR “just-in-time adaptive” OR "Cell Phones" OR mhealth OR m-health) OR TX "Mobile health" |
| S14 | S10 AND S11 AND S12 |
| S15 | S10 AND S11 AND S13 |

|  | Query Embase |
| --- | --- |
| #1 | resilience.ti,ab,kw. OR (psychological AND (resilience OR adaptation)).ti,ab,kw. |
| #2 | cognit* AND therapy OR cbt OR (cognit* AND behav* AND therapy) OR 'cognitive training' |
| #3 | cognit* AND (apprais OR reapprais OR refram OR 're fram' OR modificat OR restructur) OR ((emotion OR affect) AND (regulat OR dysregulat)) OR coping.ti,ab,kw. |
| #4 | 'reward sensitivity' OR ((positiv* OR interpret* OR cognit* OR attribut*) AND bias*.ti,ab,kw.) |
| #5 | (cognit OR affect OR behavior) AND flexibil OR (task AND switch) |
| #6 | extinction.ti,ab,kw. AND learn$.ti,ab,kw. OR (fear.ti,ab,kw. AND (condition$.ti,ab,kw. OR learn$.ti,ab,kw.)) OR (extinction AND psychological) |
| #7 | ((interference.ti,ab,kw. OR response.ti,ab,kw.) AND inhibit$.ti,ab,kw.) or ‚Inhibition (Psychology)’ or ‚inhibitory control’ |
| #8 | (stress.ti,ab,kw. OR stressor.ti,ab,kw.) AND (immunization.ti,ab,kw. OR inoculation.ti,ab,kw. OR control.ti,ab,kw. OR controllability.ti,ab,kw.) OR 'learned helplessness' OR 'situation control' |
| #9 | (‚Attentional Bias’ OR (attention$.ti,ab,kw. AND (bias$.ti,ab,kw. OR control.ti,ab,kw.))) |
| #10 | #1 or #2 or #3 or #4 or #5 or #6 or #7 or #8 or #9 |
| #11 | train$ OR interven$ OR prevent$ OR therapy OR program OR treat$. OR app |
| #12 | (ecological AND momentary AND assessment) OR (ecological.ti,ab,kw. AND momentary.ti,ab,kw.) OR smartphone.ti,ab,kw. OR telemedicine OR 'just in time' OR (mobile AND applications) OR (cell AND phone) OR mhealth.ti,ab,kw. OR 'm health.ti,ab,kw.' OR 'mobile health' |
| #13 | #10 and #11 and #12 |

|  | Query CENTRAL |
| --- | --- |
| #1 | [mh "Resilience, Psychological"] OR resilience OR [mh "Adaptation, Psychological"] |
| #2 | [mh "Cognitive Behavioral Therapy"] OR (CBT):ti,ab,kw OR (cognit* NEAR/2 behav* NEAR/2 therapy):ti,ab,kw OR (cognitive NEAR/1 training) |
| #3 | ((cognit*):ti,ab,kw AND (apprais* OR reapprais* OR refram* OR re-fram* OR modificat* OR restructur*):ti,ab,kw) OR ((emotion* OR affect*):ti,ab,kw AND (regulat* OR dysregulat*):ti,ab,kw) OR (coping):ti,ab,kw |
| #4 | reward NEAR/1 sensitivity OR ((positiv* OR interpret* OR cognit* OR attribut*):ti,ab,kw AND (bias*):ti,ab,kw) |
| #5 | ((cognit* OR affect* OR behav*) AND flexibil*) OR task NEAR/1 switch* |
| #6 | (extinction:ti,ab,kw AND learn*:ti,ab,kw) OR (fear:ti,ab,kw AND condition*:ti,ab,kw) OR (fear:ti,ab,kw AND learn*:ti,ab,kw) OR [mh "Extinction, Psychological"] |
| #7 | interference NEAR/1 inhibit* OR response NEAR/1 inhibit* OR [mh "inhibiton (Psychology)"] OR inhibitory NEAR/1 control OR [mh "executive function"] |
| #8 | ((stress OR stressor) AND (immunization OR immunisation OR inoculation OR control OR controllability)):ti,ab,kw OR learned NEAR/1 helplessness OR situation NEAR/1 control |
| #9 | [mh “attentional bias”] OR ((attention*):ti,ab,kw AND (bias* OR control):ti,ab,kw) |
| #10 | #1 OR #2 OR #3 OR #4 OR #5 OR #6 OR #7 OR #8 OR #9 |
| #11 | (train* OR interven* OR prevent* OR therap* OR program* OR app):ti,ab,kw |
| #12 | [mh "Ecological momentary assessment"] OR (Ecological AND momentary):ti,ab,kw OR [mh Smartphone] OR [mh Telemedicine] OR (smartphone OR Just-in-time NEAR/1 adaptive OR mhealth OR m-health):ti,ab,kw OR [mh "mobile Applications"] OR [mh "Cell Phone"] OR "mobile health" |
| #13 | #10 AND #11 AND #12 |
