# Supplementary material for: Cognitive reappraisal in mHealth interventions to foster mental health in adults: a systematic review and meta-analysis
Source: Front Digit Health. 2023 Oct 20;5:1253390. doi: 10.3389/fdgth.2023.1253390 (PMC10623449; doi:10.3389/fdgth.2023.1253390)
Supplement: Supplementary Material A Supplementary Material B Supplementary Material C Supplementary Material D Supplementary Material E Supplementary Material F — Full search strategy. Coding. Study characteristics. Proportion of cognitive reappraisal. Study quality assessment. Moderator analysis. [file Datasheet1.zip › B) Coding.DOCX]

**Supplementary Material B:** Coding and Data Extraction Details.

**Availability of human support**: The degree of human support, for example by a mental health professional was documented. Here, *with human support* was coded if single or group sessions, personalized face-to-face feedback (e.g., on homework exercises), or general guidance (e.g., offering help and assistance, encouragement) were provided during the training. We documented *without human support*, if training contents were exclusively provided by a mobile device and contact between the study team and the participants was only for introduction sessions (e.g., to hand-out mobile devices) or technical support.

**Additional treatment:** Furthermore, it was assessed whether the mHealth interventions were accompanied by other treatments (e.g., PC-based training, medication, face-to-face psychotherapy sessions) or stand-alone.

**Intervention triggering:** Intervention triggers were defined as follows: *on-demand* if the intervention was initiated by the participant; *triggered* if the participant was prompted to engage in intervention components; or *fixed* if the schedule of the intervention sessions was prescribed either in time (e.g., every day) or intensity (e.g., one module each week). In cases where *fixed* was coded, no additional information about whether the participants were triggered was given in the study.

**Intervention Type**: *Treatment* refers to an intervention, that focus on the reduction of symptoms of mental disorders. Both, samples with fully diagnosed mental disorder as well as samples with elevated symptoms (e.g., self-reported) are included here. *Preventions* aim at reducing the risk for the development of a mental disorder. They may address the entire population or vulnerable groups or individuals. *Mental health promotion* are interventions, that target the entire population while enhancing positive aspects of functioning and well-being.

**Proportion of cognitive reappraisal:** We included mHealth interventions in our review that at least had one component targeting cognitive reappraisal. We considered studies that used terms like cognitive restructuring, cognitive reinterpretation, reframing, or modification, as well as descriptions of the intervention content that clearly correspond to reappraisal (e.g., “establish new automatic thoughts”). We further assessed the degree to which the mHealth interventions focused on the promotion of cognitive reappraisal. Therefore, two independent reviewers extracted the components of each mHealth intervention related to cognitive reappraisal as well as components containing other contents (e.g., psychoeducation, behavioural exercises). The proportion of cognitive reappraisal was defined as the percentage of the intervention components related to cognitive reappraisal (N _CR components_/ N _all components_).
