# Supplementary material for: Cognitive reappraisal in mHealth interventions to foster mental health in adults: a systematic review and meta-analysis
Source: Front Digit Health. 2023 Oct 20;5:1253390. doi: 10.3389/fdgth.2023.1253390 (PMC10623449; doi:10.3389/fdgth.2023.1253390)
Supplement: Supplementary Material A Supplementary Material B Supplementary Material C Supplementary Material D Supplementary Material E Supplementary Material F — Full search strategy. Coding. Study characteristics. Proportion of cognitive reappraisal. Study quality assessment. Moderator analysis. [file Datasheet1.zip › C) Study characterstics.DOCX]

**Supplementary Material C.** Study characteristics.

| **First Author, Reference** | **Population** | **Symptoms of Disorders** | **Mean Age (SD)** | **% Female** | **Country** | **Intervention/Group Name ^1^** | **Compa-rator** | **Interven-tion length (in days)** | **Human support** | **Stand-alone ^2^** | **Intervention trigger(s)** | **Intervention Type ^3^** | **Interven-tion Focus** |
| --- | --- | --- | --- | --- | --- | --- | --- | --- | --- | --- | --- | --- | --- |
| Ahorsu (59) | clinical | somatic (epilepsy) | 38.2 (11.7) | 58.4 | Iran | CBT-I App | active | 42 | yes | yes | fixed, on-demand | prevention | insomnia |
| Al-Refae (60) | non-clinical | none | 25.2 (8.7) | 78.8 | Canada | Serene App | passive | 30 | no | yes | on-demand | promotion | depression, stress |
| Bakker (61) | non-clinical | none | 34.6 (11.0) | 81.0 | Australia | Moodkit | passive, active | 30 | no | yes | on-demand | promotion | mental health |
| Bruehlman-Senecal (62) | non-clinical | none | 18.7 (0.4) | 59.3 | USA | Nod | passive | 28 | no | yes | triggered, on-demand | prevention | loneliness in students |
| Bruhns (63) | non-clinical | mental (depression) | 23.0 (3.4) | 89.3 | Germany | MCT & More | passive | 28 | no | yes | triggered, on-demand | treatment | depression |
| Dagöö (64) | non-clinical | mental (SAD) | 36.8 (11.2) | 51.9 | Sweden | mCBT | active | 42 | no | yes | fixed, on-demand | treatment | anxiety (SAD) |
| Dahne (65) | non-clinical | mental (depression) | 37.7 (11.8) | 66.7 | USA | iCouch CBT | passive, active | 56 | no | yes | on-demand | treatment | depression |
| Dahne (66) | clinical | mental (depression) | 43.3 (12.9) | 84.6 | USA | Moodkit | passive, active | 56 | no | no | on-demand | treatment | depression |
| Depp (67) | clinical | mental (schizophrenia, schizoaffective or bipolar I disorder) | 49.5 (11.5) | 49.3 | USA | CBT2go | passive, active | 84 | yes | no | triggered | treatment | global psychopathology |
| Greer (68) | clinical | somatic (cancer),  mental (anxiety) | 56.4 (11.3) | 73.8 | USA | CBT mobile | active | 84 | no | yes | fixed | treatment | anxiety (cancer related) |
| **Reference** | **Population** | **Symptoms of Disorders** | **Mean Age (SD)** | **% Female** | **Country** | **Intervention/Group Name ^1^** | **Compa-rator** | **Interven-tion length (in days)** | **Human support** | **Stand-alone ^2^** | **Intervention trigger(s)** | **Intervention Type** | **Interven-tion Focus** |
| Ham (69) | clinical | somatic (cancer), mental (depression, anxiety) | 44.2 (11.0) | 85.7 | South Korea | HARUToday | passive, active | 70 | no | yes | fixed, on-demand | treatment | anxiety (cancer related) |
| Hunt (70) | clinical | somatic (IBS) | 32.0 (10.2) | 75.2 | USA | Zemedy App | passive | 56 | no | yes | fixed, triggered, on-demand | prevention | irritable bowel syndrome |
| Hur (71) | non-clinical | mental (depression) | 23.7 (3.1) | 88.2 | Korea | Todac Todac | active | 21 | no | yes | fixed | treatment | depression |
| Imamura (72)  Sasaki (73) | non-clinical | none | 33.1 (6.8) | 84.9 | Vietnam | Program A | passive | 70 | no | yes | on-demand | prevention | depression, anxiety |
| Jannati (74) | non-clinical | mental (depression) | 27.5 (4.6) | 100.0 | Iran | Happy Mom | passive | 60 | no | yes | fixed | treatment | depression (postpartum) |
| Jarvis (75) | non-clinical | none | 74.9 (6.4) | 81.3 | South Africa | mLINCC | passive | 90 | yes | no | fixed, on-demand | prevention | loneliness in elderly people |
| Liu (76) | non-clinical | mental (depression) | 23.1 (1.8) | 55.4 | China | Chatbot | active | 112 | no | yes | on-demand | treatment | depression |
| Lüdtke (77) | non-clinical | mental (depression) | 37.5 (12.7) | 75.0 | Germany | POI | passive | 28 | no | no | on-demand | treatment | depression |
| Lukas (78) | non-clinical | mental (depression) | 29.8 (9.2) | 82.0 | Germany | MT-Phoenix | passive | 14 | no | yes | on-demand | treatment | depression |
| Mantani (79, 80)  Furukawa (81)  Imai (82) | clinical | mental (depression) | 40.9 (8.9) | 53.1 | Japan | Kokoro | passive | 56 | no | no | fixed | treatment | depression |
| **Reference** | **Population** | **Symptoms of Disorders** | **Mean Age (SD)** | **% Female** | **Country** | **Intervention/Group Name ^1^** | **Compa-rator** | **Interven-tion length (in days)** | **Human support** | **Stand-alone ^2^** | **Intervention trigger(s)** | **Intervention Type** | **Interven-tion Focus** |
| McCloud (82) | non-clinical | mental (depression, anxiety) | 24.3 (6.6) | 85.1 | UK | Feel Stress Free | passive | 56 | no | yes | on-demand | treatment | depression, anxiety |
| Meyer (83) | clinical | somatic (epilepsy), mental (depression) | 40.3 (13.2) | 63.5 | Germany | Eymna | passive | 90 | no | no | on-demand | treatment | depression (in epilepsy) |
| Moberg (84) | non-clinical | mental (depression, anxiety) | 30.2 (10.9) | 54.8 | USA | Pacifica | passive | 30 | no | yes | triggered, on-demand | treatment | stress, anxiety, depression |
| Newman (85) | non-clinical | mental (GAD) | 21.4 | 77.0 | USA | Guided Self-Help | passive | 90 | yes | yes | triggered, on-demand | treatment | anxiety (GAD) |
| Oh (86) | clinical | mental (panic disorder) | 41.0 (11.8) | 51.2 | South Korea | Chatbot Group | active | 28 | no | yes | on-demand | treatment | anxiety (panic disorder) |
| Röhr (87) | non-clinical | mental (PTSD) | 33.3 (11.3) | 38.3 | Germany | Sanadak App | active | 28 | no | yes | on-demand | treatment | posttraumatic stress |
| Roepke (88) | non-clinical | mental (depression) | 40.2 (12.3) | 69.6 | USA | CBT-PPT-SuperBetter | passive, active | 28 | no | yes | on-demand | treatment | depression |
| Stiles-Shields (89) | non-clinical | mental (depression) | . | . | USA | Thought-Challenger | passive, active | 56 | yes | no | on-demand | treatment | depression |
| Stolz (90) | non-clinical | mental (SAD) | 34.9 (10.8) | 65.6 | Switzerland | ICBT App | passive | 84 | yes | yes | fixed | treatment | anxiety (SAD) |
| Zeng (91)  Zhu (92) | clinical | somatic (HIV), mental (depression) | 28.3 (5.9) | 7.7 | China | Run4Love | passive | 90 | yes | yes | fixed, triggered, on-demand | treatment | depression |

^1^ name of study group that was chosen as intervention group (mHealth intervention with cognitive reappraisal component) for the meta-analysis

^2^ It is reported whether the mHealth intervention is stand-alone or combined with additional intervention elements (e.g., coaching sessions, PC based training)

Abbreviations: SAD = social anxiety disorder, IBS =irritable bowel syndrome, GAD = generalized anxiety disorder, PTSD = post-traumatic stress disorder, HIV = human immunodeficiency virus
