# Supplementary material for: Cognitive reappraisal in mHealth interventions to foster mental health in adults: a systematic review and meta-analysis
Source: Front Digit Health. 2023 Oct 20;5:1253390. doi: 10.3389/fdgth.2023.1253390 (PMC10623449; doi:10.3389/fdgth.2023.1253390)
Supplement: Supplementary Material A Supplementary Material B Supplementary Material C Supplementary Material D Supplementary Material E Supplementary Material F — Full search strategy. Coding. Study characteristics. Proportion of cognitive reappraisal. Study quality assessment. Moderator analysis. [file Datasheet1.zip › D) Proportion of cognitive reappraisal.DOCX]

**Supplementary Material D.** Terminology and proportion of cognitive reappraisal training in the mHealth interventions.

| **Reference** | **Cognitive Reappraisal Component** | **Additional Components (N)** | **% of Cognitive Reappraisal** |
| --- | --- | --- | --- |
| Ahorsu et al. (2020) | Cognitive restructuring | 6 | 14 |
| Al-Refae et al. (2021) | Cognitive restructuring | 4 | 20 |
| Bakker et al. (2018) | Modifying thoughts | 4 | 20 |
| Bruehlman-Senecal et al. (2020) | Cognitive restructuring | 5 | 17 |
| Bruhns et al. (2021) | Cognitive reframing | 5 | 17 |
| Dagöö et al. (2014) | Cognitive restructuring | 6 | 14 |
| Dahne et al. (2019a) | Cognitive restructuring | 4 | 20 |
| Dahne et al. (2019b) | Identify alternative thoughts | 0 | 100 |
| Depp et al. (2019) | Cognitive restructuring | 5 | 17 |
| Greer et al. (2019) | Creating new thoughts | 6 | 14 |
| Ham et al. (2019) | Cognitive restructuring | 5 | 17 |
| Hunt et al. (2021) | Cognitive restructuring | 4 | 20 |
| Hur et al. (2018) | Cognitive restructuring | 0 | 100 |
| Imamura et al. (2021) | Cognitive restructuring | 4 | 20 |
| Jannati et al. (2020) | Thought challenging | 6 | 14 |
| Jarvis et al. (2019) | Restructuring of maladaptive thinking patterns | 2 | 33 |
| Liu et al. (2022) | Establish new automatic thoughts | 1 | 50 |
| Lüdtke et al. (2018) | Cognitive restructuring | 2 | 33 |
| Lukas et al. (2021) | Functional thoughts | 8 | 11 |
| Mantani et al. (2017) | Cognitive restructuring | 3 | 25 |
| McCloud et al. (2020) | Cognitive restructuring | 4 | 20 |
| Meyer et al. (2019) | Cognitive modification | 6 | 14 |
| Moberg et al. (2019) | Reframe thoughts | 9 | 10 |
| Newman et al. (2021) | Cognitive reframing | 4 | 20 |
| Oh et al. (2020) | Distorted thoughts | 5 | 17 |
| Röhr et al. (2021) | Cognitive restructuring | 2 | 33 |
| Roepke et al. (2015) | Cognitive restructuring | 3 | 25 |
| Stiles-Shields et al. (2019) | Thought restructuring | 0 | 100 |
| Stolz et al. (2018) | Cognitive restructuring | 7 | 13 |
| Zeng et al. (2022) | Thought replacement | 4 | 20 |

*Note*. All mHealth interventions included a component on cognitive reappraisal. The terminology for cognitive reappraisal of the study authors is presented. Additional components of the mHealth interventions (e.g., psychoeducation, behavioral exercises) were extracted in duplicate. The percentage of cognitive reappraisal is the proportion of the CR component in relation to all intervention components (N _CR components_/ N _all components_).
