# Supplementary material for: Cognitive reappraisal in mHealth interventions to foster mental health in adults: a systematic review and meta-analysis
Source: Front Digit Health. 2023 Oct 20;5:1253390. doi: 10.3389/fdgth.2023.1253390 (PMC10623449; doi:10.3389/fdgth.2023.1253390)
Supplement: Supplementary Material A Supplementary Material B Supplementary Material C Supplementary Material D Supplementary Material E Supplementary Material F — Full search strategy. Coding. Study characteristics. Proportion of cognitive reappraisal. Study quality assessment. Moderator analysis. [file Datasheet1.zip › F) Moderator analysis.DOCX]

**Supplementary Material F.** Results of the moderator analysis for populations with elevated symptoms of mental disorders compared to populations without symptoms of mental disorders.

| **Mental disorder symptoms** | ***n*** | ***K*** | ***M*(SMD)** | **95%CI_l_** | **95%CI_u_** | ***p*** |
| --- | --- | --- | --- | --- | --- | --- |
| Yes | 23 | 45 | 0.40 | 0.17 | 0.64 | <.001 |
| No | 7 | 17 | 0.14 | -0.21 | 0.49 | .441 |

Q_M_(1) = 1.99, *p* = .159
