# Supplementary figures and images for: Cognitive reappraisal in mHealth interventions to foster mental health in adults: a systematic review and meta-analysis
Source: Front Digit Health. 2023 Oct 20;5:1253390. doi: 10.3389/fdgth.2023.1253390 (PMC10623449; doi:10.3389/fdgth.2023.1253390)

**Supplementary Material E**. Risk of Bias Assessment (RoB2) of the included studies


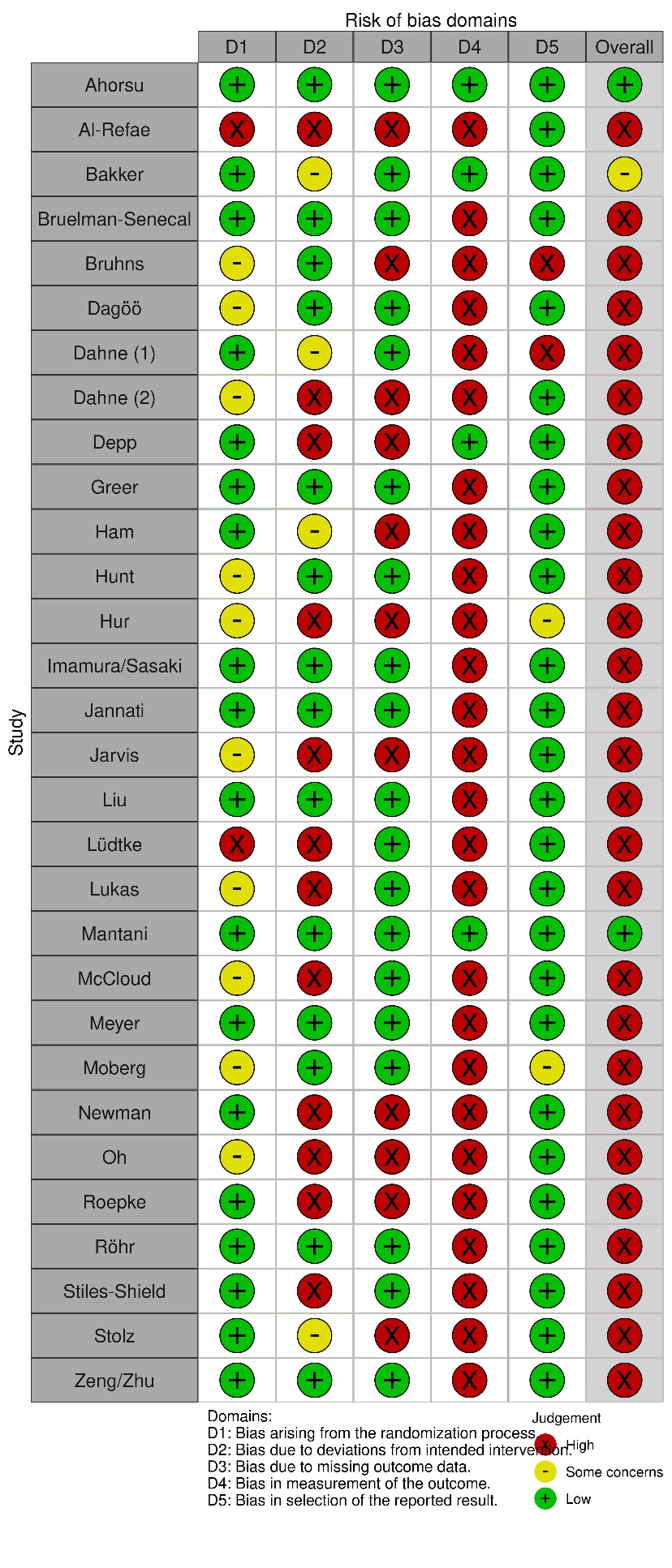

Supplement: Supplementary Material A Supplementary Material B Supplementary Material C Supplementary Material D Supplementary Material E Supplementary Material F — Full search strategy. Coding. Study characteristics. Proportion of cognitive reappraisal. Study quality assessment. Moderator analysis. [file Datasheet1.zip › E) Study quality assessment.DOCX]
